# Exploring the Antifungal Activity and Action of *Saussurea costus* Root Extracts against *Candida albicans* and Non-*albicans* Species

**DOI:** 10.3390/antibiotics11030327

**Published:** 2022-03-01

**Authors:** Melad F. Soliman, Youssria M. Shetaia, Ahmed A. Tayel, Alaa M. Munshi, Fuad A. Alatawi, Mohammed A. Alsieni, Mahmoud A. Al-Saman

**Affiliations:** 1 Department of Industrial Biotechnology, Genetic Engineering and Biotechnology Research Institute, University of Sadat City (USsC), Sadat City 22857, Egypt; microbiologistmelad@gmail.com (M.F.S.); mahmoud.alsaman@gebri.usc.edu.eg (M.A.A.-S.); 2 Department of Microbiology, Faculty of Science, Ain Shams University, Cairo 11566, Egypt; yousseriashetaia@sci.asu.edu.eg; 3 Faculty of Aquatic and Fisheries Sciences, Kafrelsheikh University, Kafrelsheikh 33516, Egypt; 4 Department of Chemistry, Faculty of Applied Science, Umm Al-Qura University, Mecca 24243, Saudi Arabia; amamunshi@uqu.edu.sa; 5 Department of Biology, Faculty of Science, University of Tabuk, Tabuk 47512, Saudi Arabia; falatawi@ut.edu.sa; 6 Department of Pharmacology, Faculty of Medicine, King Abdulaziz University, Jeddah 21589, Saudi Arabia; alsieniabdulbasiti@gmail.com

**Keywords:** anticandidal, *S. costus* roots, direct bioautography, GC/MS

## Abstract

The isolation and assessment of the active constituents in polar and non-polar crude extracts of *Saussurea costus* roots as antifungal agents, against *Candida albicans* and non-*C*. *albicans* (NAC) species, was the aim of this current investigation. The SEM “Scanning electron microscopy” imaging provided potential action modes of *n*-hexane extract (*nh*hE) toward *Candida* spp., whereas the TLC-DB “Thin layer chromatography-direct bioautography” was employed for detecting the anticandidal compounds. *nh*hE had the greatest biocidal activity against all strains and clinical isolates of *Candida* spp. with maximum zones of inhibition. SEM revealed the occurrence of irregular, dense inclusions of *C. albicans* cell walls after treatment with *nh*hE for 12 h. Complete morphological distortions with lysed membranes and deterioration signs appeared in most treated cells of *C. parapsilosis*. The most effectual compound with anticandidal activity was isolated using TLC-BD and identified as sesquiterpene by GC/MS analysis. The infra-red analysis revealed the presence of lactone ring stretching vibrations at 1766.72 cm^−1^. The anticandidal activity of *nh*hE of *S. costus* roots was confirmed from the results, and the treated cotton fabrics with *nh*hE of *S. costus* possessed observable activity against *C. albicans*. Data could recommend the practical usage of *S. costus* extracts, particularly *nh*hE, as influential natural bioactive sources for combating pathogenic *Candida* spp.

## 1. Introduction

*Candida* species are yeasts that can dangerously cause many types of fungal infections in human body/organs, whereas “Candidiasis” is the general term to categorize the infectious diseases from *Candida* contagion [1]. *Candida* species are prevalent cutaneous, vaginal, and gastrointestinal invaders. *Candida* species do not regularly cause noticeable impairment in healthy people, but they can cause a variety of diseases in particular circumstances, including chronic candidiasis, vaginitis, and endophthalmitis [2].

Infections caused by *Candida* have become much more common over the world, with fatality rates exceeding 70% in some patient groups [3]. Over 90% of *Candida*-related infections are caused by *C. albicans* and non-*C*. *albicans* (NAC) species, e.g., *C. tropicalis*, *C. parapsilosis*, *C. glabrata*, and *C. krusei* [4]. *C. auris* additionally emerged lately as a serious nosocomial pathogen that can cause invasive infections and healthcare-associated outbreaks in hospitals around the world [3,4,5].

*Candida*-caused mycoses have a variety of clinical manifestations and are classed as superficial, mucosal, or invasive candidiasis. *C. albicans* is a popular human yeast pathogen that is responsible for the majority of *Candida* genus infections, both superficial and systemic. Candidiasis is mostly of the following different types: thrush, cutaneous, vaginal, and deep candidiasis [5,6,7].

Amphotericin, azole, echinocandin, and flucytosine are the four principal types of antifungal drugs approved by the Food and Drug Administration (FDA) of the United States [8]. Antifungal agents such as echinocandins and azoles are the most effective against candidiasis, and echinocandins have less adverse effects than other antifungal groups, but many *Candida* species can develop resistance to them [9,10,11]. Despite the efficacy of these antifungal classes in treating yeast and mold infections, many fungal pathogens have acquired resistance to them [12].

The antifungal resistance involves multifactorial complex phenomena that are not fully understood and haven’t been fully elucidated; resistance to antifungal drugs could develop as a result of mutations in the fungus’ DNA, which lead to alterations in the pathogen’s drug metabolism; the other potential mechanisms involving *Candida* spp. resistance (especially biofilm forming cells) include elevated efflux pump potentiality, excessive cells’ density within biofilm, consequences of biofilm matrix, nutrient and growth limitations, the persister cells occurrence, or increased sterols contents on cells’ membranes [2,8,10]. The molecular antifungal mechanisms also include genomic plasticity; resistance to chromosomes’ duplication; mini-chromosomes formation that are harboring copious number of resistance genes; appearance of genes alleles with hyperactivity; mis-translation of tRNAs codons; and heterozygosity loss [2,3,8,10].

*Saussurea costus* is used in Indian medical systems as a solo medicine or in conjunction with other pharmaceuticals. Its roots have antispasmodic, antifungal, and antibacterial properties and are used to treat asthma, cough, cholera, chronic skin illnesses, and rheumatism; this advocates *S. costus* to be utilized in further human-related healthcare and remedies [13,14,15].

Due to the exponential rise in drug-resistant infections and the expensive cost of therapeutics, medicinal plants with antifungal activity could be prospective sources of novel antimicrobial agents in the form of pure components or crude extracts [16]. This experimental study aimed to isolate and investigate the active constituents in several organic crude extracts from the roots of *S. costus* and evaluate their bioactivities against *C. albicans* and NAC species.

## 2. Materials and Methods

### 2.1. Plant Collection, Identification and Preparation

The dried roots of identified *Saussurea costus* plants were attained from ARC-EG “Agricultural Research Center—Giza, Egypt”. After washing the roots of *S. costus* with double distilled water (DDW), the roots were air-dried for 72 h. The dried roots were pulverized with electric grinder and then stored in the brown screw-cap bottles at −18 °C.

### 2.2. Crude Extracts Preparation and Fractionation Procedures

A pulverized root sample (1.850 kg) was extracted with 95% methanol (3 L) and was agitated for 48 h at room temperature (RT) using a rotary shaker (150 rpm). To remove root particles, in a Buchner funnel, the extract was filtered using filter paper Whatman No. 1 [17,18].

**Figure A1:**
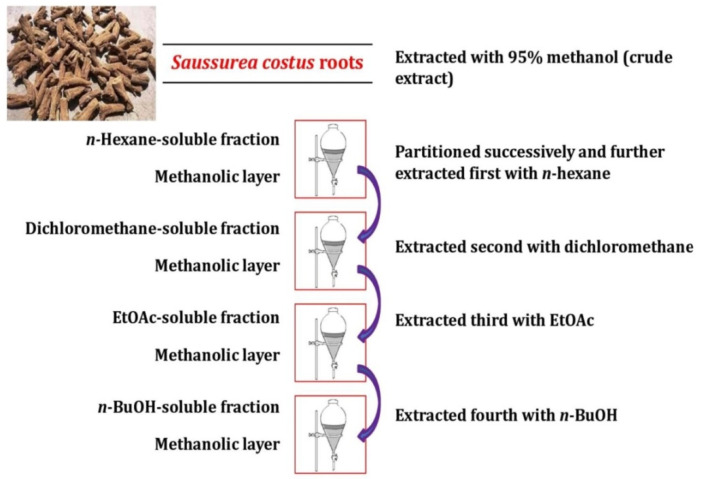
*Saussurea costus* root crude extraction and solvent-solvent fractionation scheme.

Components of the methanolic extract were separated by partitioning in solvents of different polarities. The separation of those components was carried out sequentially between the methanol mixture and increasing polarity solvents: *n*-hexane, *n*-butanol (*n*-BuOH), dichloromethane, and ethyl acetate (EtOAc) [19,20], (Figure A1).

### 2.3. Standard Candida Strains

In this study, reference strains of five *Candida* species, including *C. tropicalis* (Seq. ID: Cand-Mar.1-EG2018; Acess No: MH 445555), *C. parapsilosis* (Seq. ID: Cand-Mar.12-EG2018; Acess no: MH 445556), *C. pseudotropicalis* (Biochemical ID), and *C. guillimondii* (Biochemical ID) were obtained from Animal Health Research Institute, Cairo, Egypt, and *C. albicans* (ATCC 10231) were obtained from Epico, Egyptian Pharmaceutical Company, Cairo, Egypt. The identities of entire standard strains were confirmed with molecular identification methods (e.g., PCR, real-time PCR and MALDI-TOF), as illustrated from providing institutions (the nucleotide sequence alignments of ITS genes of identified *Candida* spp. with other *Candida* spp. strains are provided in Supplementary Materials). Thus, these strains served as the standards for comparisons with external isolates.

To ensure purity, viability, and pathogenicity, the strains were kept at −70 °C in sterile distilled water with 50% glycerol and were sub-cultured on Sabouraud dextrose agar (SDA, Merck, Germany), chromogenic *Candida* agar (CHROMagar^TM^ Candida media, CHROMagar, Paris, France), and blood agar (CM0031, Oxoid, Basingstoke, UK) at 37 °C for 24 h.

### 2.4. Isolation and Identification of Clinical Isolates

In the present study, 30 clinical isolates were isolated from urine, vaginal swabs, and wounds. The clinical isolates were cultured on SDA augmented with chloramphenicol and were aerobically incubated at 37 °C for 48 h.

Other procedures, such as Gram stain, germ tube test, and culture on chromogenic *Candida* agar were performed after direct microscopic examinations and sub-culturing on SDA [21]. The clinical isolates were identified by classical methods and were compared to standard strains, as the main target of study was to evaluate the anticandidal activity of extracted biomolecules against diverse *Candida* spp. However, more identification methods using molecular biology techniques (e.g., 18S rRNA) are intended for future analysis of *Candida* strains (Supplementary Materials).

### 2.5. Screening of Anticandidal Activity

The anticandidal activity of the crude extracts was tested using the traditional agar well diffusion method for all *Candida* strains and isolates. Each *Candida* isolate and strain was suspended in a sterile saline solution (5 mL) and was adjusted to a transmittance (T) of 75–77% at 530 nm, which equaled to 1 × 10^6^ CFU/mL.

To evenly streak SDA plates, a cotton swab dipped in inoculum suspension was employed. The 6-mm diameter filter paper discs (Whatman No. 41) were placed on top of inoculated agar and impregnated with 25 µL extract produced with 20% Tween 80 in distilled water at a concentration of (2 mg/mL) [22].

Plates were incubated for 48 h at 37 °C. The diameter of the newly appeared zone of inhibition (ZOI) was then precisely measured, and the means of the triplicates were determined. Anticandidal discs containing fluconazole (2 mg/mL) (Diflucan injection, Pfizer, New York, NY, USA) was placed on the inoculated medium as a positive control. After incubating the media at 37 °C for 24 h, the zone of inhibition around the disc was measured. Tween 80 (20%) was used to make the negative control. Each extract was tested against the examined *Candida* strains and isolates as a quantitative experiment to determine minimal inhibitory concentrations (MIC) [23].

### 2.6. Scanning Electron Microscopy (SEM)

In this work, the *n*-hexane extract (*nh*hE) has a strong anticandidal effect against *Candida* strains. For this reason, the morphological changes in the two different strains of *Candida* cells (*Candida albicans*, ATCC 10231 and *Candida parapsilosis*, MH 445556) were visualized and were confirmed using SEM within 6 and 12 h after treatment with *nh*hE of *S. costus* roots at a concentration of (2 mg/mL).

The cells were collected by centrifugation at 6000 rpm for 15 min at 4 °C, then were washed with phosphate-buffered saline (PBS, Sigma, St. Louis, MO, USA; pH 7.4), and fixed with 2% glutaraldehyde and 1% osmium tetroxide (OsO_4_). The samples were rinsed in PBS and dehydrated in escalating order of ethanol concentrations after fixation. The preserved candidal cells were then thoroughly dried before being covered with a thin layer of gold. Under a SEM (JSM 5300 SEM; JEOL, Tokyo, Japan), all samples were examined [24].

### 2.7. Chromatographic Fractionation of the n-Hexane Extract of S. costus

#### 2.7.1. Thin Layer Chromatography

On silica gel plates, the main components of the crude *nh*hE were qualitatively identified (Merck, silica gel 60F-254 TLC aluminum sheet 20 × 20 cm). Using the Drummond micro capillary tube on the plate, 20 µL of 5% *n*-hexane fraction diluted with methanol was manually spotted.

The screening program used a variety of mobile phases with varying concentrations (Table 1). After complete solvent evaporation, the separated compounds were visualized with a UV lamp (365 and 254 nm) and developed with a vanillin/H_3_PO_4_ spray reagent (the composing materials were attained from Sigma-Aldrich, St. Louis, MO, USA) before being heated to 110 °C [25]. Different spots with R*_f_* values were measured and calculated.

#### 2.7.2. Thin Layer Chromatography-Direct Bioautographic (TLC-DB) Assay

A bioautographic assay was utilized to determine the active components, as described by [26]. TLC was used to separate the samples first, and then the inoculation medium was added to the developed TLC plates to detect the active bands. Two hundred microliters of candidal suspension (*as described above*) were prepared and spread on the created TLC plate with a sterilized glass rod until just moist. For the sake of minimizing other airborne germs, in aseptic conditions under laminar flow the plate inoculated with the target organism was allowed to dry. The TLC plate was then incubated at 37 °C for 24–36 h. For contrast, fluconazole was used as a conventional antifungal drug. Anticandidal compounds were recognized as clear inhibition zones against a background of non-inhibition that indicated candidal growth. A duplicate chromatogram was created under the same conditions and compared to the results of the experiment.

#### 2.7.3. Flash Chromatography Separation

At Nawah Scientific Laboratory in Egypt (www.nawah-scientific.com, accessed on 23 January 2022), semi-preparative separation of the active molecules from the crude *nh*hE was performed using a PFCC4100 system (Interchim; Montluçon, France), which included a mixing HPLC quaternary pump, a photo diodarray (PDA)—UV-Vis detector 190–840 nm, a fraction collector, and a sample loading module. Interchim software 5.0 was utilized for system control and process monitoring. Medium pressure liquid chromatography (MPLC) separations were carried out on a plastic column (80 g—flash—NP column (30 μm) 20 bar Interchim PF-30SHIP-JP-F0080). Using 50 g silica as a dry load, 10 g of crude *nh*hE was injected into the column. *Mobile phase*: petroleum ether (solvent A), ethyl acetate (solvent B); 0–100% B gradient in 240 min at 20 mL/min flow rate.

The fractions that showed the elution of comparable compounds based on TLC technique were pooled and concentrated for 30 min under vacuum at temperatures below 40 °C. The active fraction was also analyzed using high-performance liquid chromatography (HPLC) (depending on anticandidal activity).

#### 2.7.4. Gas Chromatography–Mass Spectrometry (GC/MS) Analysis

GC/MS analysis was performed on the crude *nh*hE, PFCC pooled fractions, and active fraction individually to determine the volatile components based on their retention duration and peak area using a Thermo Scientific, Trace GC Ultra/ISQ Single Quadrupole MS, TG-5MS fused silica capillary column (30 m, 0.251 mm, 0.1 mm film thickness).

A percent relative peak area was used to evaluate the quantification of all the discovered components. The compounds’ relative retention times and mass spectra were compared to those of the NIST, WILLY library data of the GC/MS system to make a preliminary identification.

#### 2.7.5. Infrared Spectra

After preparative TLC separation and anticandidal activity determination, the isolated active compound obtained from PFCC was analyzed using a FTIR instrument (SHIMADZU, IR Spirit) at Ahram Canadian University, Central Research Lab., Giza, Egypt; in the frequency range of 4000 to 400 cm^−1^, and the results were compared to a reference of typical IR absorptions.

### 2.8. Anticandidal Action of Cotton Fabric Wound Dressings Treated with nhhE of S. costus

The cotton wound dressings utilized in this investigation (Style S/400, 106 g/m^2^ plain texture, TESTEX, Essen, Germany) were scoured and sterilized. Cotton wound dressing pieces were soaked in *nh*hE of *S. costus* at its MIC level, at pH 6.5 0.2, for 2 h at 60 °C, with stirring. The treated fabrics were dried at 65 °C with hot air for 5 min before being cured at 25 ± 2 °C in the lab. The anticandidal activity of fabrics treated with *nh*hE was evaluated qualitatively using an agar well diffusion assay. A 2 × 2 cm sector of untreated (negative control) and *nh*hE-treated textiles were put on the surface of SDA plates, which were inoculated with *C. albicans* (ATCC 10231) for 36 h at 37 °C. Anticandidal activity was indicated by the formation of a growth-free zone surrounding the cotton fabric [27].

### 2.9. Statistical Analysis

Using SPSS version 17.0 statistical software, the experimental data were reported as the mean SD of three replicates (IBM Corporation, Armonk, NY, USA). One-way analysis of variance (ANOVA) and Duncan’s multiple range tests were used to compare means for in vitro antibacterial assessment. Statistical significance was distinct as a *p* value of less than 0.05.

## 3. Results

### 3.1. Extraction and Fractionation

The results revealed a considerable difference in extraction yield when various solvents were used. Methanol had the highest extraction yield (58.5%), followed by *n*-hexane (9.0%), dichloromethane (5.0%), *n*-BuOH (2.7%), and EtOAc (1.0%), suggesting that highly polar solvents have a highest extraction efficiency (Figure A1).

### 3.2. Candida Species Identification

**Figure A2:**
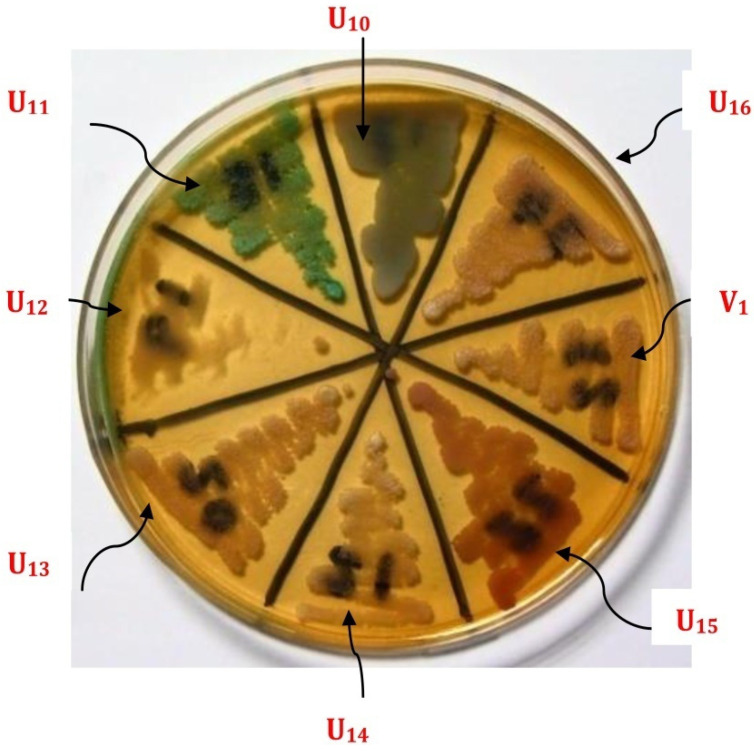
Model for the growth of some isolates after 48 h on CHROM agar. (U_10_) blue to gray colonies (*C. tropicalis*); (U_11_) green colonies (*C. albicans*); (U_12,13,14,16_ and V_1_) off-white colonies (*C. parapsilosis*); (U_15_) pink colonies (*C. glabrata*).

The employment of CHROMagar medium for differentiating *Candida* spp. indicated that the growth of *Candida tropicalis* appeared as (blue to grey), *Candida albicans* as (green), *Candida parapsilosis* as (off-white), and *Candida glabrata* as (pink) colonies after 48 h on CHROMagar (Figure A2). *C. albicans* (43.34%) was the most common isolated *Candida* species, followed by *C. parapsilosis* (30%), *C. glabrata* (10%), *C. tropicalis* (10%), *C. pseudotropicalis* (3.33%), and *C. guillimondii* (3.33%).

### 3.3. Anticandidal Activity of Saussurea costus Root Extracts

*S. costus* root extracts were obtained using a variety of solvents and were tested for anticandidal activity against a variety of standard *Candida* strains (5 strains) and clinical *Candida* isolates (30 isolates). The presence of inhibitory zones diameters was used to determine the anticandidal activity qualitatively. As shown in results of Table 2, the crude extracts of *n*-hexane and dichloromethane were effective against different strains and isolates of *Candida*, except for EtOAc, *n*-BuOH, and methanol extracts that had no inhibitory effect against any strain or isolate.

*Candida pseudotropicalis* is the most resistant strain of *Candida* to all extracts, except for *nh*hE. It showed the lowest inhibition zone that was followed by *C. tropicalis isolates*. Whereas, isolates U_10_, U_13_, U_14_, and U_16_ were the most resistant *Candida* isolates to *n*-hexane and dichloromethane extracts, except for *nh*hE, with the lowest inhibition zone followed by isolates U_7_, U_8_, U_25_, and U_26_. On the other hand, isolates no. U_5_ and U_24_ were the most sensitive isolates with the highest inhibition zone scored.

The crude *nh*hE showed the maximum inhibition of growth against isolates U_5_, U_9_, U_13_, and U_24_ (18, 17, 17, and 18 mm, respectively) at 2 mg/mL (as standard concentration used for all extracts). Less inhibition was observed against isolates W_2_ and U_23_ (10 mm). Whereas, with dichloromethane extract, maximum inhibition of growth (12 mm) was recorded against *C. guillimondii* biochemical ID strain, and *candida* isolates U_5_ U_11_, and U_24_, Also, less inhibition of growth (0.8 mm) with dichloromethane extract was recorded against *C. pseudotropicalis* biochemical ID strain and *Candida* isolates U_10_, U_13_, U_14_, and U_16_.

Dichloromethane was the least efficient extract against all *Candida* standard strains and clinical isolates of *Candida*. In comparison to the conventional antifungal employed at the same dose, *n*-hexane was the most efficient solvent used for extraction of *S. costus* roots in case of all *Candida* standard strains and clinical isolates of *Candida*, since it gave the greatest zone inhibition.

### 3.4. Scanning Electron Microscopy (SEM)

**Figure 1:**
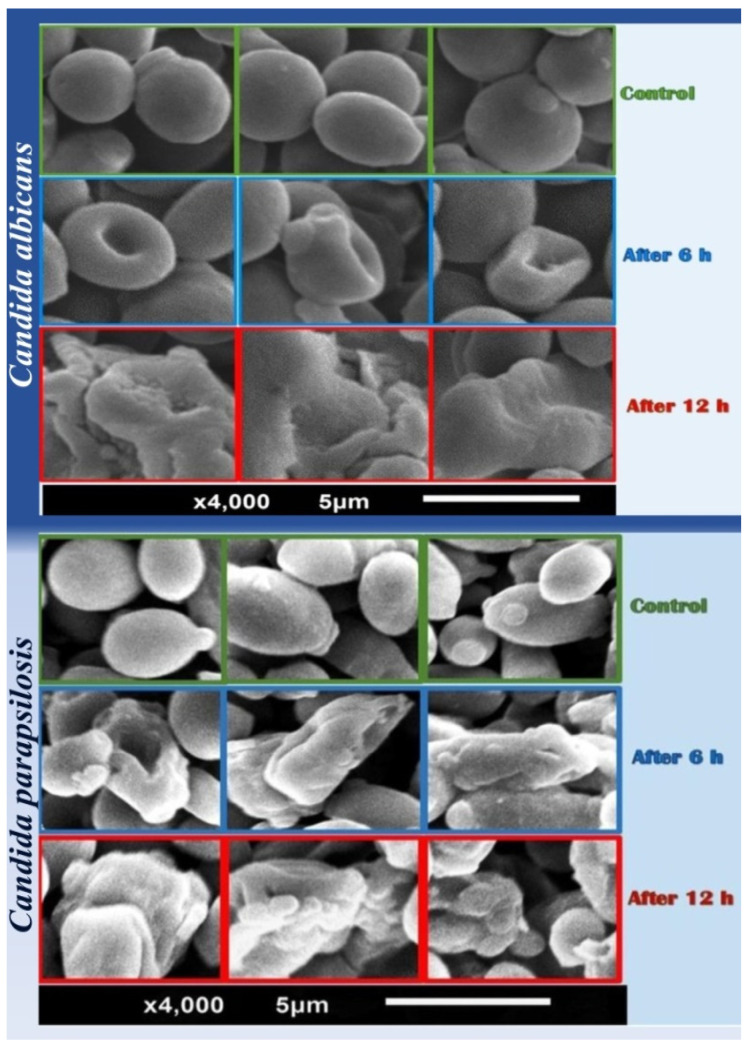
SEM images of un-treated and treated *Candida albicans* (ATCC 10231) and *Candida parapsilosis* (MH 445556) with *n*-hexane extract of *S. costus* after exposure for 6 h and 12 h.

*C. albicans* (ATCC 10231) blasto spores in SEM were mainly smooth-walled structures with a spherical to elongated shape in control cells. All of the yeast cells were lying side by side, with polar buds and bud scars visible (Figure 1-*Candida albicans*-Control). The surface micromorphology of treated cells was altered in the majority of the studied cells.

After 6 h at a concentration of 2 mg/mL, the surface of cells treated with *nh*hE revealed a well-defined wrinkling of the cell wall. In addition, the yeast cells were seen in groups of interconnected cells. (Figure 1-*Candida albicans*-after 6 h).

The deposition of irregular, dense inclusions in walls and sections of the cell wall that protruded into the cytoplasm after treatment with *nh*hE for 12 h was noticed and was regarded as an indicator of wall collapse (Figure 1-*Candida albicans*-after 12 h).

SEM was also performed to find out the morphological changes in *C. parapsilosis* (MH 445556) after exposure to *S. costus* *nh*hE at a concentration of (2 mg/mL) for 6 and 12 h.

Untreated cells showed spherical cells with smooth surface (Figure 1-*Candida parapsilosis*-Control), whereas treated cells showed complete morphological changes with an elongated surface, rough appearance, and most of the cells had completely deteriorated after 12 h by the release of cellular constituents, which indicated cytoplasmic damage of cells (Figure 1-*Candida parapsilosis*-after 6 and 12 h).

### 3.5. Chromatographic Fractionation of Anticandidal Substances of Crude n-Hexane Extract

#### 3.5.1. Thin-Layer Chromatography (TLC)

**Figure 2:**
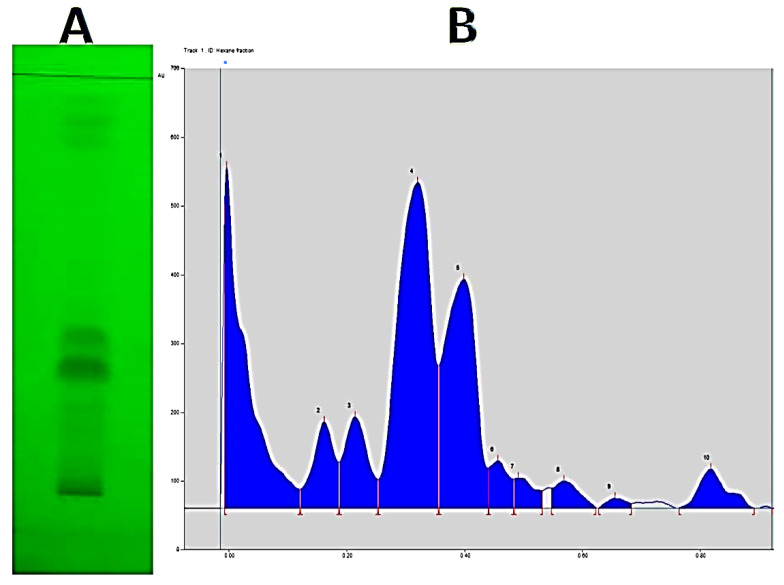
UV illumination at 365 nm (**A**) of a normal TLC plate developed in a solvent mixture of petroleum ether: EtOAc (9.3:0.7, *v/v*) and Scanning of TLC chromatogram (**B**) using CAMAG TLC Scanner.

In this study, the best solvent for extracting the active anticandidal compounds from the *S. costus* roots was *n*-hexane. TLC analysis with a solvent mixture of petroleum ether: EtOAc (9.3:0.7, *v/v*) revealed 10 distinct bands, 2 of which were unique and had retention factor (R*_f_*) values of 0.30 and 0.40 (Figure 2A).

TLC chromatogram scanning (CAMAG TLC Scanner 3) validated the TLC results, and R*_f_* values of all compounds were matched with those of TLC (Figure 2B).

#### 3.5.2. Thin Layer Chromatography-Direct Bioautography (TLC-DB)

**Figure A3:**
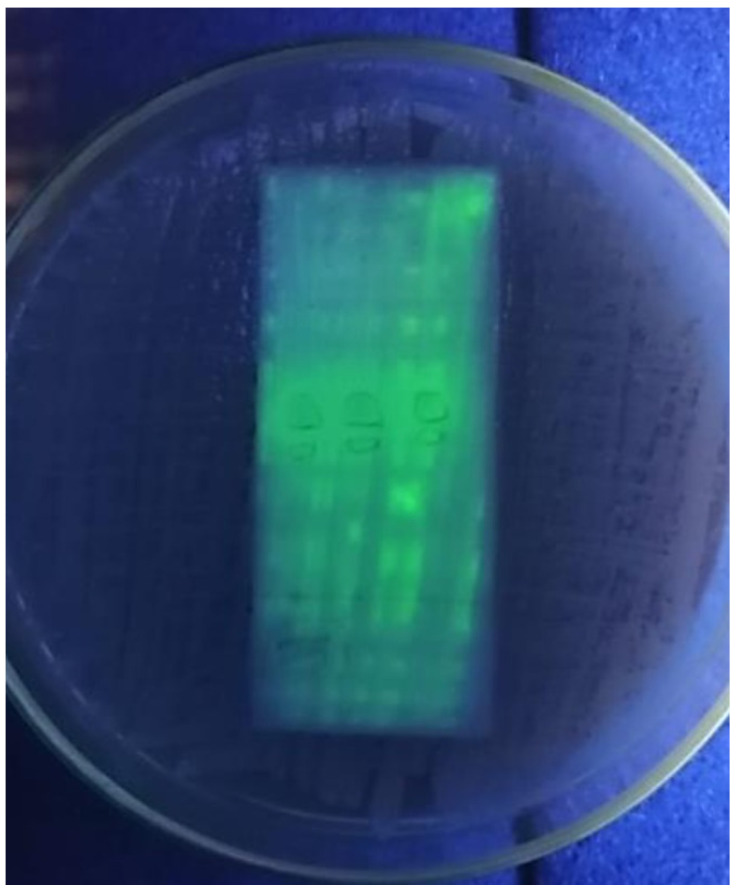
TLC-DB with anticandidal activity, developed in a solvent mixture of petroleum ether: EtOAc (9.3:0.7, *v/v*) and examined under UV at 365 nm.

A TLC-DB technique was used to detect anticandidal compounds in a crude *nh*hE of *S. costus*. The growth of *Candida* was inhibited in the presence of anticandidal compounds in the *nh*hE applied to the chromatogram, resulting in the development of a clear inhibition zone at R*_f_* value 0.40 (band No. 5) against the background of the non-inhibition zone on the chromatogram (Figure A3).

#### 3.5.3. Purification and Identification of the Anticandidal Compounds

In an attempt to isolate and characterize the active compound (s), *nh*hE obtained from *S. costus* roots was fractionated using PFCC. One-hundred-and-seventy-six fractions were collected using a fraction collector, and anticandidal activity was investigated against different standard strains of *Candida* and clinical *Candida* isolates. Positive anticandidal activities were found in fractions managed from 120 to 130 with similar R*_f_* values based on the TLC technique. The pooled fraction was monitored second on TLC, which was developed using petroleum ether: ethyl acetate (93:7, *v/v*) and was observed in a UV chamber at 365 nm. The TLC profile showed a single band at the position of R*_f_* value 0.40.

#### 3.5.4. GC/MS Analysis

**Figure 3:**
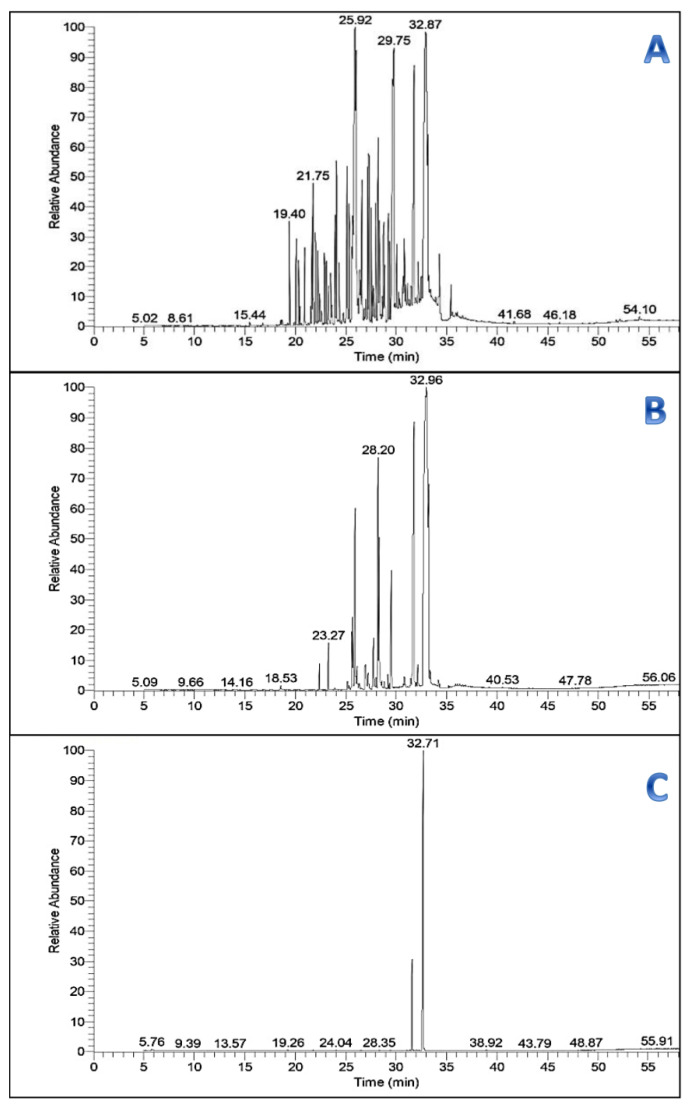
GC/MS chromatograms of (**A**) the crude *n*-hexane extract of *S*. *costus*; (**B**) PuriFlash 4100 system all bold fractions; and (**C**) active fraction (individually).

The volatile components of an *n*-hexane extract of *Saussurea costus* roots were analyzed by GC–MS. Fifty volatile compounds were identified and classified as sesquiterpenes, diterpenes, and triterpenes based on their chemical structures. The essential extract contained six major sesquiterpenes: α-selinene (2.27%), caryophyllene oxide (2.12%), 8-cedren-13-ol (3.04%), costunolide (germacranolide) (5.38%), cis-lanceol (6.56%), and dehydrocostuslactone (22.5%); as well as two major diterpenes and triterpenes ethyl linoleate (0.39%) and α-amyrin (0.69%), respectively. Other volatile compounds also detected included 11,14,17-eicosatrienoic acid methyl ester (11.40%); 1-acetyl-6-methylbenzotriazole (6.27%); hexadecanoic acid, ethyl ester (palmitic acid ethyl ester) (0.36%); and 9,12-octadecadienoic acid (z,z)-, methyl ester (0.90%) (Figure 3A and Table 3).

The PFCC pooled fractions upon GC-MS analysis showed several peaks with sesquiterpene (dehydrocostus lactone, C_15_H_18_O_2_) possessing the highest percentage of area normalization (54.23%); the mass spectrum was found to have a probability (>59.83) with that of the authentic compound from the GC-MS library (Figure 3B). The bioactive fraction upon GC-MS analysis showed a major peak with the same active constituent dehydrocostus lactone (at the same previous retention times) possessing the highest percentage of area normalization (83.48%; at a concentration equivalent to 1.437 g/kg) (Figure 3C). Based on the GC-MS analysis, the active fraction was structurally elucidated as dehydrocostus lactone. The analysis reports revealed the presence of dehydrocostuslactoneas as an effective anticandidal agent extracted from the *n*-hexane extract of *S. costus*.

**Figure 4:**
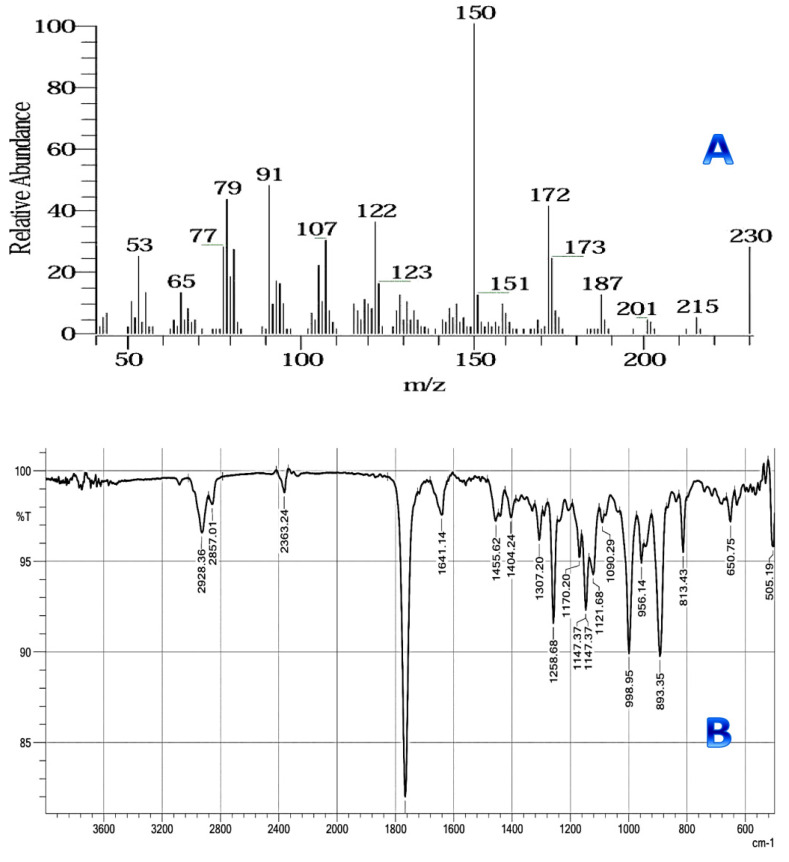
Mass spectrum (**A**) and Infrared spectrum (**B**) of the isolated anticandidal compound “dehydrocostus lactone” from the *n*-hexane roots extract of *Saussurea costus*.

**Figure 5:**
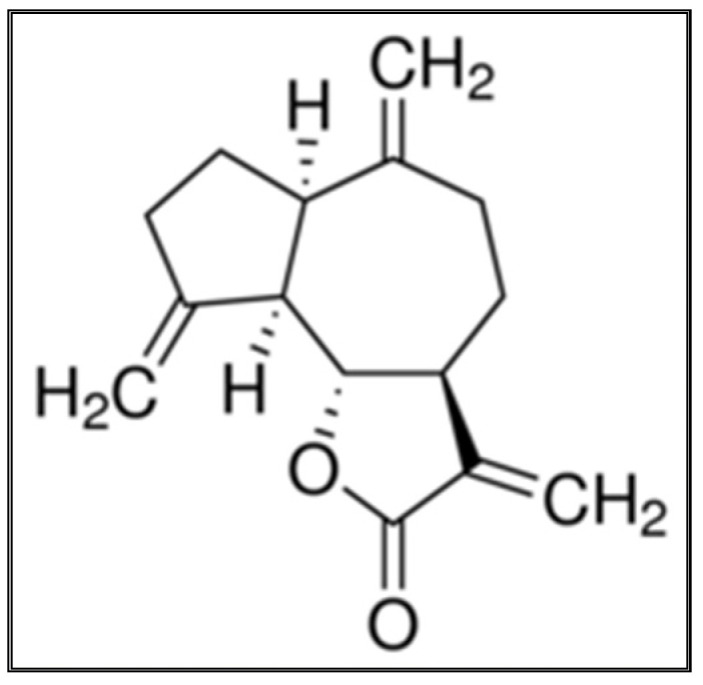
Formula for the structure of a dehydrocostus lactone.

The mass spectrum fragmentation pattern of the active compound under investigation is shown (Figure 4A). It revealed the presence of major molecular ion peaks at *m*/*z* 91, 150, and 172 of relative abundance characteristic of the parent compound dehydrocostus lactone (Figure 5).

#### 3.5.5. Characterization of the Isolated Compound

The result in Figure 4B indicated the isolated compound’s infrared (IR) spectrum. The functional and/or structural groupings’ absorption peaks were recorded.

The stretching vibrations of the C-H aliphatic and aromatic C-H groups exhibited a band at 2928.36 cm^−1^ in the IR spectrum. A broad strong band appeared at 1766.72 cm^−1^, which signified the presence of a stretching vibration of lactone ring containing an oxygen atom. There were also bands due to the stretching vibration of the C-O group at 1258.68 cm^−1^.

### 3.6. Assessment of Anticandidal Activity of n-Hexane Extract Using Cotton Wound Dressing

**Figure 6:**
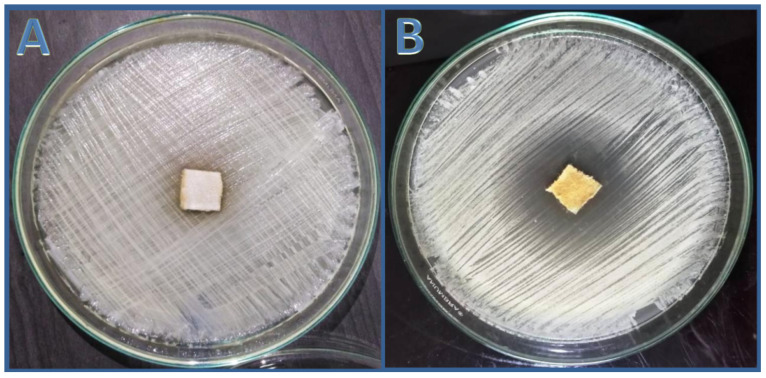
Anticandidal activity of untreated (**A**) and treated (**B**) cotton fabrics with *n*-hexane extract of *S. costus* against *C. albicans* (ATCC 10231) after 36 h.

The cotton fabric was treated with an *nh*hE derived from the *S. costus* roots at a MIC of (200 g/mL), and the cotton fabric demonstrated substantial anticandidal activity via agar diffusion assay (Figure 6B), but the untreated fabric showed no anticandidal activity against *C. albicans* (ATCC 10231) (Figure 6A). After extending the incubation time for up to 7 days, the formed clear zone encircling the treated sector liberated active constituents of the extract into the agar medium and inhibition of the entire *C. albicans* cells without the occurrence of any resistant mutations to *S. costus* *nh*hE.

## 4. Discussion

*Saussurea costus* is commonly known as costus or the Indian costus [28]. *S. costus* has been used as a remedy for thousands of years, and the root of the plant is the main part used for therapeutic purposes and was recommended by Prophet Muhammad “treat with the Indian incense, for it has healing for seven diseases” [29]. Falconer was the first to show that *S. costus* roots, among other species, were utilized by ancient physicians and more lately by Japanese scientists for cancer treatment, and that *S. costus* extracts and tinctures have been included in many pharmacopoeias [30]. The US FDA considers *S. costus* to be generally recognized as safe (GRAS) when taken as a dietary supplement.

In this study, the anticandidal activity of the extracts of *S. costus* root was examined. *nh*hE of *S. costus* showed a significant anticandidal activity, while *n*-BuOH and EtOAc extracts did not inhibit the growth of any tested *Candida*; *nh*hE inhibited the growth of most *C. albicans* and NAC species such as *C. tropicalis*, *C. parapsilosis*, *C. pseudotropicalis*,d an *C. guillimondii*.

*S. costus* root extracts have been shown to significantly inhibit human pathogenic bacteria and fungi recently in in vitro investigations. Using the “cup plate diffusion” method, the petroleum ether, chloroform, methanol, and water extracts of *S. lappa* (synonym for *S. costus*) were tested against Gram^+^ and Gram^−^ bacteria; the chloroform extract yielded the best results [31]. *S. costus* extracts, according to [32], can play a significant role in the defense action against human multi-resistant bacteria and can be used instead of antibiotics in the treatment of specific diseases. The current effectiveness of *S. costus* phytochemicals in controlling several *C. albicans* and NAC species advocates their applicability for combating other serious emerging species like *C. auris*.

Thin layer chromatography-direct bioautography bioassay has potential applications such as the rough quantification of the antimicrobial compound, as the radius of the zone of inhibition is proportional to the logarithm of the amount of antimicrobial compound [33]. TLC plate bioassays also served to narrow the range of possible anticandidal compounds in the *nh*hE.

According to Asokkumar and Ramachandran [34], the essential oil of *S. lappa* roots has a larger number of components based on gas chromatography (GC) and mass spectrometry. Monoterpenoids make up a smaller amount of the essential oil (14%) than sesquiterpenoids (80%). The primary components of *S. lappa* essential oil were dehydrocostus lactone (46%) and costunolide (10%). The lower concentration of dehydrocostus lactone (22.5%) obtained in this study compared to other results may be due to the plant’s nature and its environment.

Sesquiterpenes are a type of terpene made up of three isoprene units that can be acyclic or ringed and come in a variety of forms. Sesquiterpene lactones are a type of sesquiterpene with a lactone ring, as the name suggests. They were found in many plant families, such as Acanthaceae, Amaranthaceae, and Costaceae, and can cause toxicity [35]. The results obtained agreed with the formerly obtained results [17,32], which found that the main components isolated from *S. costus* roots are sesquiterpene lactones such as costunolide and dehydrocostus lactone, which have antibacterial, antifungal, and cytotoxic effects.

Low polar sesquiterpene lactones have more powerful antifungal activity [13]. It is well established that the occurrence of α-methylene-γ-lactone is required for sesquiterpene lactones to have significant antifungal activity. These compounds’ low polarity corresponds to the ideal lipophilic properties needed to pass through the fungal cell wall [36]. In addition, sesquiterpene lactones have the optimal polarity level for antifungal action [13]. This could be linked to the degree of lipophilicity necessary for sesquiterpenoids to pass through the fungal cell wall. The results obtained agreed with former reports that *S. costus* species are rich in dehydrocostus lactone, a sesquiterpene compound that acts as a selective inhibitor and could be potentially used as an ingredient of a new medication [37]. Dehydrocostus lactone has a good anticandidal effect, according to the findings of our investigation.

Lactones, particularly sesquiterpenoids, have biological activities due to their inhibitory effects on a variety of enzymes found in bacterial organisms and eukaryotic cells. Different transcription factors are inhibited by sesquiterpene lactones [38].

Sesquiterpenoids extracted from the *Xylopia brasiliensis* leaves demonstrated efficacy against *Cladosporium cladosporioides*, according to Moreira et al. [39]. At the lowest concentrations, the isolated compound dehydrocostus lactone substantially suppressed pathogenic *Candida*. Costunolide, a compound isolated from the bark of *Magnolia grandiflora*, was found to have antifungal activity against plant pathogenic fungi [36]. The chemical compounds in costus essential oil were identified and found that *n*-hexadecanoic acid was the main constituent in all the examined essential oils accompanied with other fatty acids; hydrocarbons; and mono-, di- and sesquiterpenes [28].

Infrared and UV spectroscopy identified eremanthine as α,β-unsaturated ɤ-lactone. The chemical formula was determined to be C_15_H_18_O_2_, (mol wt 230.13) by high resolution mass spectrometry, and hydrogenation produced a hexahydro derivative that was identical to a dehydrocostus lactone (guaianolidesesquiterpene lactone; molwt 230.30) [40].

Dehydrocostus lactone, a guaianolide originally derived from *S. lappa*, can be produced through chemical transformations of eremanthine [41].

The application of antimicrobial agents to textiles and dressings is limited by several restrictions, for example, these substances should be efficient, harmless to the skin, and environment benign [42]. The original *nh*hE of the *S. costus* roots could be employed as an active ingredient in anticandidal treatments, especially for diabetics and cancer patients with chronic wounds. The SEM observations of treated *Candida* spp. with current extract phytochemicals provided potential modes of action for destructing and deforming yeast cells. However, from the proposed anticandidal actions of plant derivatives are the restraint of yeast biofilm formation and hyphal transition as well as the inhibition of (1,3)-β-D-glucan synthase (1,3)-β-D-glucan biosynthesis, forming channels with membranes ergosterol that cause leakage of cytoplasmic components, induction of cell wall deficiency and instability, and obstructing the biosynthesis of cell structural components (e.g., sphingolipids, mannoproteins and chitin) and pores’ formation in the yeast bilayer membranes [43,44]. Generally concluded, the phytochemicals anticandidal actions are mostly membranes- and walls-active mechanisms [43].

From the attained results, *S. costus* root phytochemicals are recommended for control and management of *C*. *albicans* and NAC infections. Further investigations could be suggested for overcoming the potential shortages in current study, e.g., molecular identification of isolated yeast strains using modern approaches such as PCR-based methods, relevant genes sequencing, RT-PCR, and MALDI-TOF, as these molecular methods provide more accurate and rapid *Candida* genotyping and specification [45]; assessing the minimal inhibitory (MIC) and minimal fungicide (MFC) concentrations of extract fractions and probable synergistic actions of these fractions through fractional inhibitory concentrations (FIC), fractional inhibitory concentration index (FICI) assessments, and their relationship with the reference drugs [46]; and also the specification of potential mechanisms of action using immunological assays such as membrane assays, immunofluorescence, and immunomodulatory assays. Although the biosafety and biocompatibility of most plant derivatives could be warranted, more confirming assays regarding the biotoxicity, hemolytic activity, and biocompatibility of *S. costus* extract are suggested.

## 5. Conclusions

The research goals were the isolation and identification of anticandidal compounds from the roots of *S. costus*. A bioactive compound was isolated from *n*-hexane extract of the *S. costus* roots, which exhibited anticandidal activity. TLC-DB is a powerful tool for isolating anticandidal compounds. The isolated anticandidal compound revealed asesquiterpene compound after further analysis. The IR spectra revealed the existence of functional groups that may be responsible for the component’s biological action. It was concluded that *S. costus* roots is rich in some bioactive phytochemical compounds, which makes them a promising plant for drug development research. The effectiveness of *S. costus* phytochemicals in controlling several *Candida* spp. suggests their applicability for combating more species like *C. auris*. The plant root extract of *S. costus* will, in the future, be a promising tool for drug development in this era of multiple drug-resistant pathogens.

## Figures and Tables

**Table 1:** Development of thin layer chromatography using different operating solvent systems.

Solvent Mixture	% (*v/v*)
petroleum ether: ethyl acetate	9:1
petroleum ether: ethyl acetate	10:1
petroleum ether: ethyl acetate	9.3:0.7
*n*-hexane: ethyl acetate	6.7:3.3
petroleum ether: dichloromethane: ethyl acetate	7:2.3:0.7

**Table 2:** Anticandidal activity of *n*-hexane and dichloromethane extracts of *Saussurea costus* against *Candida* strains and isolates.

Code No.	Name of the Standard Strains *Candida* and Isolates	Diameter of Zone of Inhibition (mm)				Standard Antibiotic (Fluconazole)
		*n*-Hexan		Dichloromethane		
		Zone of Inhibition	MIC (mg/mL)	Zone of Inhibition	MIC (mg/mL)	
ATCC 10231	*C. albicans*	13 ± 2	1	10 ± 1	3	10 ± 2
MH 445555	*C. tropicalis*	12 ± 2	1	0.9 ± 1	4	15 ± 2
MH 445556	*C. parapsilosis*	13 ± 2	1	11 ± 2	3.5	13 ± 2
Biochemical ID	*C. pseudotropicalis*	15 ± 2	0.25	0.8 ± 1	4.5	09 ± 2
Biochemical ID	*C. guillimondii*	14 ± 1	1	12 ± 1	3	12 ± 2
* W1	*(C. tropicalis)*	12 ± 2	1	10 ± 1	3	11 ± 2
W2	*(C. parapsilosis)*	10 ± 1	1.5	8 ± 1	3	-
** V1	*(C. parapsilosis)*	11 ± 2	1	10 ± 1	3.5	12 ± 2
V2	*(C. albicans)*	13 ± 2	1.5	10 ± 1	4	-
*** U1	*(C. albicans)*	12 ± 2	1	10 ± 1	3	13 ± 2
U2	*(C. albicans)*	11 ± 2	1.5	10 ± 1	4	12 ± 2
U3	*(C. albicans)*	12 ± 3	1	11 ± 1	3.5	14 ± 2
U4	*(C. albicans)*	13 ± 2	1	10 ± 1	3	13 ± 2
U5	*(C. albicans)*	18 ± 2	0.2	12 ± 2	3	17 ± 2
U6	*(C. albicans)*	11 ± 1	1	0.9 ± 1	3	10 ± 2
U7	*(C. albicans)*	12 ± 2	1	0.9 ± 1	3	13 ± 2
U8	*(C. parapsilosis)*	13 ± 2	1	11 ± 2	4	12 ± 2
U9	*(C. albicans)*	17 ± 2	0.2	12 ± 2	3	13 ± 2
U10	*(C. tropicalis)*	15 ± 2	0.5	0.8 ± 1	4.5	-
U11	*(C. pseudotropicalis)*	14 ± 1	0.5	12 ± 1	3	15 ± 2
U12	*(C. guillimondii)*	14 ± 3	0.5	11 ± 2	3	13 ± 2
U13	*(C. albicans)*	17 ± 1	0.2	0.8 ± 1	3	-
U14	*(C. glabrata)*	11 ± 1	1.5	0.8 ± 1	4	12 ± 2
U15	*(C. tropicalis)*	14 ± 2	0.5	11 ± 1	4	13 ± 2
U16	*(C. albicans)*	11 ± 1	1.5	0.8 ± 1	4.5	12 ± 2
U17	*(C. parapsilosis)*	13 ± 2	1	10 ± 1	3	14 ± 2
U18	*(C. parapsilosis)*	12 ± 2	1	10 ± 1	4.5	13 ± 2
U19	*(C. parapsilosis)*	12 ± 2	1	10 ± 1	4	11 ± 2
U20	*(C. glabrata)*	12 ± 3	0.5	11 ± 1	3	10 ± 2
U21	*(C. parapsilosis)*	13 ± 2	0.5	10 ± 1	3	12 ± 2
U22	*(C. parapsilosis)*	12 ± 2	1	10 ± 1	4	11 ± 2
U23	*(C. albicans)*	10 ± 1	1.5	8 ± 1	3.5	09 ± 2
U24	*(C. albicans)*	18 ± 2	0.2	12 ± 2	3	16 ± 2
U25	*(C. albicans)*	11 ± 1	1.5	0.9 ± 1	4	10 ± 2
U26	*(C. glabrata)*	12 ± 2	1	0.9 ± 1	3	13 ± 2

* W_1_–W_2_: Candida species isolated from wounds; ** V_1_–V_2_: Candida species isolated from vaginal swabs; *** U_1_–U_26_: Candida species isolated from urine.

**Table 3:** Volatiles composition of *n*-hexane extract of Saussurea costus roots.

Compound Name	Retention Time (min)	Molecular Formula	m/z Fragments ^a^	Peak Area (%)
α-Selinene	21.75	C_15_H_24_	204, 105 *, 107, 93, 79	02.27
Caryophyllene oxide	24.07	C_15_H_24_O	93, 79, 43, 41 *	02.12
11,14,17-Eicosatrienoic acid methyl ester	25.92	C_21_H_36_O_2_	79 *, 67, 55, 41	11.40
α-Amyrin	26.46	C_30_H_50_O	218 *, 203	00.69
8-Cedren-13-ol	28.20	C_15_H_24_O	119 *, 105, 91, 41	03.04
1-acetyl-6-methylbenzotriazole	29.65	C_9_H_9_N_3_O	147 *, 119, 103	06.27
Costunolide	29.73	C_15_H_20_O_2_	109, 81 *, 53, 41	05.38
cis-Lanceol	31.76	C_15_H_24_O	93 *, 91, 79, 43	06.56
Hexadecanoic acid, ethyl ester (Palmitic acid ethyl ester)	32.47	C_18_H_36_O_2_	101, 88 *, 43	00.36
dehydrocostuslactone	32.89	C_15_H_18_O_2_	150, 91 *	22.50
9,12-Octadecadienoic acid (Z,Z)-, methyl ester	34.26	C_19_H_34_O_2_	81, 67 *, 55, 41	00.90
Ethyl linoleate	35.42	C_20_H_36_O_2_	95, 81, 67 *, 55	00.39

^a,^* indicates base peak, which is the most intense ion (tallest peak).

## Data Availability

The data presented in this study are available on reasonable request from the corresponding author.

## Supplementary Materials

The following supporting information can be downloaded at: https://www.mdpi.com/article/10.3390/antibiotics11030327/s1, Supplementary File: Nucleotide sequence alignment of ITS genes of Cand-Mar.2-EG2018/Cand-Mar.1-EG2018 with other *Candida* spp. Strains.
