# Supplementary material for: Exploring the Antifungal Activity and Action of Saussurea costus Root Extracts against Candida albicans and Non-albicans Species
Source: Antibiotics (Basel). 2022 Mar 1;11(3):327. doi: 10.3390/antibiotics11030327 (PMC8944531; doi:10.3390/antibiotics11030327)
Supplement: Supplementary file 1 [file antibiotics-11-00327-s001.zip › antibiotics-1581555-supplementary.pdf]

Nucleotide sequence alignment of ITS genes of **Cand-Mar.2-EG2018** with other Candida spp. strains

```
                           ---------+---------+---------+---------+---------+
                                    10        20        30        40        50
                           ---------+---------+---------+---------+---------+
Cand-Mar.2-EG2018          AATAGAAAAACACTTGTGTAAGAAAAAATGCAGTTGAGCACTTTTCTTTC         50
C.parapsilosis-76          ..A..........A.....................A..........A...         50
C.parapsilosis-LEMI7686    ..A..........A.....................A..........A...         50
C.parapsilosis-79          ----------------...................A..........A...         34
C.parapsilosis-ZB012       ---------....AG....................A..........A...         41
C.parapsilosis-Para-CC-105 --------------------------------------------------          0
C.parapsilosis-CBS2193     --------------------------------------------------          0
C.orthopsilosis-LEM134     --------------------------------------------------          0
C.parapsilosis-CMC_1826    --------------------------------------------------          0

                           ---------+---------+---------+---------+---------+
                                    60        70        80        90       100
                           ---------+---------+---------+---------+---------+
Cand-Mar.2-EG2018          T--GTGGTCCTCTTTCCGAAGGT-GAACCTGCGGAAGGATCATTACAGAA         97
C.parapsilosis-76          .--..AA.GA..C.....T....-..........................         97
C.parapsilosis-LEMI7686    .--..AA.GA..C.....T....T..........................         98
C.parapsilosis-79          .--..AA.GA..-.....T...G-..........................         80
C.parapsilosis-ZB012       CTAAAA.GGAAAC.....T..AG-AG........................         90
C.parapsilosis-Para-CC-105 ------------......T....-..........................         37
C.parapsilosis-CBS2193     ------------......T....-..........................         37
C.orthopsilosis-LEM134     ----------------..T....-.G........................         33
C.parapsilosis-CMC_1826    --------------....C....-TC....A...................         35

                           ---------+---------+---------+---------+---------+
                                   110       120       130       140       150
                           ---------+---------+---------+---------+---------+
Cand-Mar.2-EG2018          TGAAAAGTGCTTAACTGCATTTTTTCTTACACATGTGTTTTTCTTTTTTT        147
C.parapsilosis-76          ..................................................        147
C.parapsilosis-LEMI7686    ..................................................        148
C.parapsilosis-79          ..................................................        130
C.parapsilosis-ZB012       ..................................................        140
C.parapsilosis-Para-CC-105 ..................................................         87
C.parapsilosis-CBS2193     ..................................................         87
C.orthopsilosis-LEM134     ....-....................T--......................         80
C.parapsilosis-CMC_1826    ....-....................T.-......................         83

                           ---------+---------+---------+---------+---------+
                                   160       170       180       190       200
                           ---------+---------+---------+---------+---------+
Cand-Mar.2-EG2018          --GAAAACTTTGCTTTGGTAGGCCTTCTATATGGGGCCTGCCAGAGATTA        195
C.parapsilosis-76          --................................................        195
C.parapsilosis-LEMI7686    --................................................        196
C.parapsilosis-79          --................................................        178
C.parapsilosis-ZB012       --................................................        188
C.parapsilosis-Para-CC-105 --................................................        135
C.parapsilosis-CBS2193     --................................................        135
C.orthopsilosis-LEM134     TT.................G....C-------AT................        123
C.parapsilosis-CMC_1826    TT.................G....C-------AT................        126

                           ---------+---------+---------+---------+---------+
                                   210       220       230       240       250
                           ---------+---------+---------+---------+---------+
Cand-Mar.2-EG2018          AACTCAACCAAATTTT-ATTTAATGTCAACCGATTATTTAATAGTCAAAA        244
C.parapsilosis-76          ................-.................................        244
C.parapsilosis-LEMI7686    ................-.................................        245
C.parapsilosis-79          ................-.................................        227
C.parapsilosis-ZB012       ................-.................................        237
C.parapsilosis-Para-CC-105 ................-.................................        184
C.parapsilosis-CBS2193     ................-.................................        184
C.orthopsilosis-LEM134     ................-......-......T.....AC............        171
C.parapsilosis-CMC_1826    ................T......-......T.....AC............        175
```

```
                                  ---------+---------+---------+---------+---------+
                                           260       270       280       290       300
                                  ---------+---------+---------+---------+---------+
Cand-Mar.2-EG2018                 CTTTCAACAACGGATCTCTTGGTTCTCGCATCGATGAAGAACGCAGCGAA        294
C.parapsilosis-76                 ..................................................        294
C.parapsilosis-LEMI7686           ..................................................        295
C.parapsilosis-79                 ..................................................        277
C.parapsilosis-ZB012              ..................................................        287
C.parapsilosis-Para-CC-105        ..................................................        234
C.parapsilosis-CBS2193            ..................................................        234
C.orthopsilosis-LEM134            ..................................................        221
C.parapsilosis-CMC_1826           ..................................................        225

                                  ---------+---------+---------+---------+---------+
                                           310       320       330       340       350
                                  ---------+---------+---------+---------+---------+
Cand-Mar.2-EG2018                 ATGCGATAAGTAATATGAATTGCAGATATTCGTGAATCATCGAATCTTTG        344
C.parapsilosis-76                 ..................................................        344
C.parapsilosis-LEMI7686           ..................................................        345
C.parapsilosis-79                 ..................................................        327
C.parapsilosis-ZB012              ..................................................        337
C.parapsilosis-Para-CC-105        ..................................................        284
C.parapsilosis-CBS2193            ..................................................        284
C.orthopsilosis-LEM134            ..................................................        271
C.parapsilosis-CMC_1826           ..................................................        275

                                  ---------+---------+---------+---------+---------+
                                           360       370       380       390       400
                                  ---------+---------+---------+---------+---------+
Cand-Mar.2-EG2018                 AACGCACATTGCGCCCTTTGGTATTCCAAAGGGCATGCCTGTTTGAGCGT        394
C.parapsilosis-76                 ..................................................        394
C.parapsilosis-LEMI7686           ..................................................        395
C.parapsilosis-79                 ..................................................        377
C.parapsilosis-ZB012              ..................................................        387
C.parapsilosis-Para-CC-105        ..................................................        334
C.parapsilosis-CBS2193            ..................................................        334
C.orthopsilosis-LEM134            ..................................................        321
C.parapsilosis-CMC_1826           ..................................................        325

                                  ---------+---------+---------+---------+---------+
                                           410       420       430       440       450
                                  ---------+---------+---------+---------+---------+
Cand-Mar.2-EG2018                 CATTTCTCCCTCAAACCCTCGGGTTTGGTGTTGAGCGATACGCTGGGTTT        444
C.parapsilosis-76                 ..................................................        444
C.parapsilosis-LEMI7686           ..................................................        445
C.parapsilosis-79                 ..................................................        427
C.parapsilosis-ZB012              ..................................................        437
C.parapsilosis-Para-CC-105        ..................................................        384
C.parapsilosis-CBS2193            ..................................................        384
C.orthopsilosis-LEM134            .................T................................        371
C.parapsilosis-CMC_1826           .................T................................        375

                                  ---------+---------+---------+---------+---------+
                                           460       470       480       490       500
                                  ---------+---------+---------+---------+---------+
Cand-Mar.2-EG2018                 GCTTGAAAGAAAGGCGGAGTATAAACTAATGGATAGGTTTTTTC--CACT        492
C.parapsilosis-76                 ............................................--....        492
C.parapsilosis-LEMI7686           ............................................--....        493
C.parapsilosis-79                 ............................................--....        475
C.parapsilosis-ZB012              ............................................--....        485
C.parapsilosis-Para-CC-105        ............................................--....        432
C.parapsilosis-CBS2193            ............................................--....        432
C.orthopsilosis-LEM134            ..........................................T-C....        420
C.parapsilosis-CMC_1826           ..........................................TTC....        425
```

```
                          ---------+---------+---------+---------+---------+
                                  510       520       530       540       550
                          ---------+---------+---------+---------+---------+
Cand-Mar.2-EG2018         CATTGGTACAAACTCCAAAACTTCTTCCAAATTCGACCTCAAATCAGG-A           541
C.parapsilosis-76         ................................................-.           541
C.parapsilosis-LEMI7686   ................................................T.           543
C.parapsilosis-79         ................................................T.           525
C.parapsilosis-ZB012      ................................................T.           535
C.parapsilosis-Para-CC-105 ...............................................T.           482
C.parapsilosis-CBS2193    ................................................T.           482
C.orthopsilosis-LEM134    ....................-...........................T.           469
C.parapsilosis-CMC_1826   ....................-...........................T.           474

                          ---------+---------+---------+--
                                  560       570       580
                          ---------+---------+---------+--
Cand-Mar.2-EG2018         GGACTACCCGCTGAACTTAAGCATATCATTAA                             573
C.parapsilosis-76         ................................                             573
C.parapsilosis-LEMI7686   ..............................                               573
C.parapsilosis-79         ............................A...                             557
C.parapsilosis-ZB012      ............................A...                             567
C.parapsilosis-Para-CC-105 ................................                            514
C.parapsilosis-CBS2193    ............................A...                             514
C.orthopsilosis-LEM134    ............................A...                             501
C.parapsilosis-CMC_1826   ............................A...                             506
```

Nucleotide sequence alignment of ITS genes of **Cand-Mar.1-EG2018** with other Candida spp. strains

```
                              ---------+---------+---------+---------+---------+-
                                      10        20        30        40        50
                              ---------+---------+---------+---------+---------+-
Cand-Mar.1-EG2018             CTTCCGGTAAGGGTGCCTGCGGAAGGATCAATACTGATTTGCTTAATTGCA          51
C.tropicalis-C9               ......-..G.T.AA...............T....................          50
C.tropicalis-YZ27             ......-..G.T.AA...............T....................          50
C.tropicalis-YZ1              ......-..G.T.AA...............T....................          50
C.tropicalis                  ......-..G.T.AA...............T....................          50
C.tropicalis-PHB5             ---------------......-........T....................          35
C.sojae                       ----------------..............T....................          35
C.labiduridarum-ATCC-MYA-4368 ---------------...............T....................          36
C.viswanathii-CBS4024         ---------------...............T....................          36
C.aquae_textoris-ATCC201456   ---------------...............T..A.................          36
C.saraburiensis-CBS11696      ---------------...............T....................          36

                              --------+---------+---------+---------+---------+--
                                     60        70        80        90       100
                              --------+---------+---------+---------+---------+--
Cand-Mar.1-EG2018             CCACATGTGTTTTTTATTGA---ACAAATTT-CTTTGGTGGCGGGAGC---          95
C.tropicalis-C9               ....................---........-................---          94
C.tropicalis-YZ27             ....................---........-................---          94
C.tropicalis-YZ1              ....................---........-................---          94
C.tropicalis                  ....................---........-................---          94
C.tropicalis-PHB5             ....................---........-...............----          78
C.sojae                       ..................A.TTA.A......G......C...A....ATAA          86
C.labiduridarum-ATCC-MYA-4368 ..............CT.--.TGA.......-G......CA.T...GATTTT          84
C.viswanathii-CBS4024         ................C---TGG...GC.--G......C..T...GA.TCG          82
C.aquae_textoris-ATCC201456   ................C---TGG...GG.--G......C..T...GA.TCG          82
C.saraburiensis-CBS11696      ..............CG.--TTTG.AC.GC.-G......C..G.C..TTTCG          84

                              -------+---------+---------+---------+---------+---
                                   110       120       130       140       150
                              -------+---------+---------+---------+---------+---
Cand-Mar.1-EG2018             -AATCCCACCGCCAGAGGTTATAACTAAACCAAACTTTTTATTT-AC--AG         142
C.tropicalis-C9               -.....T.....................................-..--..         141
C.tropicalis-YZ27             -.....T.....................................-..--..         141
C.tropicalis-YZ1              -.....T.....................................-..--..         141
C.tropicalis                  -.....T.....................................-..--..         141
C.tropicalis-PHB5             -.....T..........-..........................-..--..         124
C.sojae                       A...T.T.....................................T..--..         135
C.labiduridarum-ATCC-MYA-4368 --T..TTG.T...........A........A...........A.T..TT..         133
C.viswanathii-CBS4024         --T.T..G...........C.C....................--T..C-..         128
C.aquae_textoris-ATCC201456   --T.T..G...........C.C....................--T..C-..         128
C.saraburiensis-CBS11696      --G..G.G.............C....................-.C.ACG..         132

                              ------+---------+---------+---------+---------+----
                                  160       170       180       190       200
                              ------+---------+---------+---------+---------+----
Cand-Mar.1-EG2018             TCAAACTTGATTTATTATTACAA--TAGTCAAAACTTTCA--ACAACGGAT         189
C.tropicalis-C9               .......................--...............--.........         188
C.tropicalis-YZ27             .......................--...............--.........         188
C.tropicalis-YZ1              .......................--...............--.........         188
C.tropicalis                  .......................--...............CA.........         190
C.tropicalis-PHB5             .......................--...............--.........         171
C.sojae                       .......................AA..............--A.........         184
C.labiduridarum-ATCC-MYA-4368 ....-TAC..-......CC.-..--...............--.........         177
C.viswanathii-CBS4024         ....-.-CAT-ACG..T.---..--...............--.........         169
C.aquae_textoris-ATCC201456   ....-.-CAT-ACG..T.---..--...............--.........         169
C.saraburiensis-CBS11696      ....-..GAT.A....TA.....--...............--.........         178
```

```
                              -----+---------+---------+---------+---------+-----
                                  210       220       230       240       250
                              -----+---------+---------+---------+---------+-----
Cand-Mar.1-EG2018             CTCTTGGTTCTCGCATCGATGAAGAACGCAGCGAAATGCGATACGTAATAT        240
C.tropicalis-C9               ...................................................        239
C.tropicalis-YZ27             ...................................................        239
C.tropicalis-YZ1              ...................................................        239
C.tropicalis                  ...................................................        241
C.tropicalis-PHB5             ...................................................        222
C.sojae                       ...................................................        235
C.labiduridarum-ATCC-MYA-4368 ...................................................        228
C.viswanathii-CBS4024         ...............................................G...        220
C.aquae_textoris-ATCC201456   ...............................................G...        220
C.saraburiensis-CBS11696      ...............................................G...        229

                              ----+---------+---------+---------+---------+------
                                 260       270       280       290       300
                              ----+---------+---------+---------+---------+------
Cand-Mar.1-EG2018             GAATTGCAGATATTCGTGAATCATCGAATCTTTGAACGCACATTGCGCCCT        291
C.tropicalis-C9               ...................................................        290
C.tropicalis-YZ27             ...................................................        290
C.tropicalis-YZ1              ...................................................        290
C.tropicalis                  ...................................................        292
C.tropicalis-PHB5             ..G.....................G..........................        273
C.sojae                       ...................................................        286
C.labiduridarum-ATCC-MYA-4368 ...................................................        279
C.viswanathii-CBS4024         ........................A..........................        271
C.aquae_textoris-ATCC201456   ...................................................        271
C.saraburiensis-CBS11696      ...................................................        280

                              ---+---------+---------+---------+---------+-------
                                310       320       330       340       350
                              ---+---------+---------+---------+---------+-------
Cand-Mar.1-EG2018             TTGGTATTCCAAAGGG-CATGCCTGTTTGAGCGTCATTTCTCCCTCAAACC        341
C.tropicalis-C9               ................-..................................        340
C.tropicalis-YZ27             ................-..................................        340
C.tropicalis-YZ1              ................-..................................        340
C.tropicalis                  ................-..................................        342
C.tropicalis-PHB5             ................G..................................        324
C.sojae                       ................-..................................        336
C.labiduridarum-ATCC-MYA-4368 ................-..................................        329
C.viswanathii-CBS4024         ................-...............................G..        321
C.aquae_textoris-ATCC201456   ................-...............................G..        321
C.saraburiensis-CBS11696      ................-...............................G..        330

                              --+---------+---------+---------+---------+--------
                               360       370       380       390       400
                              --+---------+---------+---------+---------+--------
Cand-Mar.1-EG2018             CCCGGGTTTGGTGTTGAGCAATACGCTA-GGTTTGTTTGAAAGAATTTAAC        391
C.tropicalis-C9               ............................-......................        390
C.tropicalis-YZ27             ............................-......................        390
C.tropicalis-YZ1              ............................-......................        390
C.tropicalis                  ............................-......................        392
C.tropicalis-PHB5             ............................-......................        374
C.sojae                       .TT.........................-...............-.G....        385
C.labiduridarum-ATCC-MYA-4368 ..T.........................C...............CG.--..        378
C.viswanathii-CBS4024         .G........................C.-...............CG.--..        369
C.aquae_textoris-ATCC201456   .G......................C.C.-.............A.CG.--..        369
C.saraburiensis-CBS11696      .G.........................TC...............TG.--..        379

                              -+---------+---------+---------+---------+---------
                              410       420       430       440       450
                              -+---------+---------+---------+---------+---------
Cand-Mar.1-EG2018             GTGGAAACTTATTTT-AAGCGACTTAGGTTTATCCAAAAACGCTTATTTTG        441
C.tropicalis-C9               ...............-...................................        440
C.tropicalis-YZ27             ...............-...................................        440
C.tropicalis-YZ1              ...............-...................................        440
C.tropicalis                  ...............-...................................        442
C.tropicalis-PHB5             ...............T..C.............C......-...........        424
C.sojae                       ..........--.C.T.............................TG...A        434
C.labiduridarum-ATCC-MYA-4368 .....TTT.-T.-A.TT..............T......---A...T---GC        421
C.viswanathii-CBS4024         .....G...-..-A.T-..............CTA....---A.GCT---..        411
C.aquae_textoris-ATCC201456   .........-..-A.T-.C............CTA....---A.GCT---G.        411
C.saraburiensis-CBS11696      ....G...G-.CCA.G..A............CTA....CG.A.G.T.CAGC        429
```

```
                              +---------+---------+---------+---------+---------+
                              460       470       480       490       500       510
                              +---------+---------+---------+---------+---------+
Cand-Mar.1-EG2018             C-TAGTGGCCACCACAATTTATTTCATAAACTTTGACCTCAAATAAGGTAG        491
C.tropicalis-C9               .-...........................-..............C......        489
C.tropicalis-YZ27             .-...........................-..............C......        489
C.tropicalis-YZ1              .-...........................-..............C......        489
C.tropicalis                  .-...........................-..............C......        491
C.tropicalis-PHB5             .-.....-..-..............CAT................C......        472
C.sojae                       --...............C..T...---..CT.............C..-...        479
C.labiduridarum-ATCC-MYA-4368 TT.TA.A.AAG....T..AAC...T.A..CT.............C......        472
C.viswanathii-CBS4024         TGC...C.G.-....C.CAGC...TC...CT.--..........C......        459
C.aquae_textoris-ATCC201456   TGC...C.G.-....C.CAGC...TC...CT.............C......        461
C.saraburiensis-CBS11696      TGCG.CC.A.G....C.CAAC..C....-CT.............C......        479

                              ---------+---------+---------+---------+-
                                       520       530       540       550
                              ---------+---------+---------+---------+-
Cand-Mar.1-EG2018             GACTACCCGCTGAACTTAAGCATATCAATAAGCGG-GGAAA                  531
C.tropicalis-C9               ...................................A.-...                  529
C.tropicalis-YZ27             ...................................A.....                  530
C.tropicalis-YZ1              ...................................A.....                  530
C.tropicalis                  ...................................A.....                  532
C.tropicalis-PHB5             ...................................A.....                  513
C.sojae                       .................................C.G                       515
C.labiduridarum-ATCC-MYA-4368 ...................................                        507
C.viswanathii-CBS4024         ...............................A...                        494
C.aquae_textoris-ATCC201456   ..................................                         495
C.saraburiensis-CBS11696      ............................                               507
```
